# Influence of parental rules about screen electronic device use in the evening on sleep in adolescents

**DOI:** 10.1186/s12982-025-00923-w

**Published:** 2025-09-06

**Authors:** Kevin Mammeri, Laura Riontino, Sophie Schwartz, Virginie Sterpenich

**Affiliations:** 1 Department of Basic Neurosciences, University of Geneva, Geneva, Switzerland; 2 Swiss Center for Affective Science, University of Geneva, Geneva, Switzerland

**Keywords:** Adolescents, Screen electronic devices, Parental rules, Sleep, Academic achievement

## Abstract

**Purpose:**

Sleep is essential for effective daily cognitive and affective functioning, both of which are critical in the school context. In recent years, average nighttime sleep duration has been decreasing, particularly among teenagers, in parallel with an increase in screen time. Here, we aimed at assessing whether parental rules pertaining to the use of electronic devices in the evening were associated with enhanced sleep duration in healthy adolescents.

**Methods:**

We asked 329 adolescents (13–15 years old) to fill in questionnaires collected within their schools.

**Results:**

We show that only the strictest parental rules regarding screen use (no smartphone in the room and no phone use in the evening) correlated with significantly longer sleep duration. Finally, our investigation revealed that sleep duration was linked to academic achievement, which was further enhanced by the presence of parental rules.

**Discussion:**

Taken together, these results suggest that promoting greater parental control over the use of electronic devices may potentially help alleviating sleep loss in adolescents.

**Supplementary Information:**

The online version contains supplementary material available at 10.1186/s12982-025-00923-w.

## Introduction

Sleep quality and quantity influence daytime cognitive and emotional functioning. Experimental studies demonstrated that sleep contributes to memory consolidation [1, 2], vigilance [3], and emotional balance [4]. Conversely, chronic sleep disruption (e.g., insomnia, delayed sleep phase) has been associated with increase in negative mood states and earlier psychiatric disorders onset like anxiety or depression [5]. Alarmingly, several studies comparing pre and during/post COVID-19 pandemic revealed that sleep duration in adolescents was lower before the pandemic, due to early awakenings related to school, below the 8 to 10 h of sleep per night recommended for teenagers aged between 13 and 18 years old [6–12]. A recent review confirmed that delaying school start times increased sleep duration in adolescents by allowing them to align with their natural biological circadian shift associated with pubertal development [13]. Gariepy et al. [14] confirmed this decrease of sleep duration in adolescents across most of the 24 European and North American countries investigated. Relatedly, chronic sleep restriction in adolescents is more severe and pervasive nowadays than over the past decades [15, 16], with up to 70% of adolescents reporting sleep problems (usually insomnia, nocturnal awakenings, and delayed sleep phase syndrome), which were also found to be associated with poorer academic performance [17]. Academic performance is influenced by numerous factors, including economic status, parental monitoring, teacher support which then may be mediated through different variables such as motivation, self-efficacy and sleep parameters [18, 19]. Notably, a meta-analysis identified small but significant associations between daytime sleepiness and poorer academic performance in adolescents [20]. Given that academic achievement is a key contributor to life satisfaction during adolescence and plays a critical role in shaping future career and job opportunities, it is essential to examine this variable in relation to sleep habits during this developmental stage [21].

Phones, computers, tablets, televisions are omnipresent in adolescents’ daily lives. According to reports from the nonprofit organization “Common Sense” adolescents aged between 13 and 18 years old, in the USA, spent on average seven and a half hours per day on screen electronic devices in 2019 (without including time dedicated to school or homework), rising to eight and a half hours per day during the COVID-19 pandemic [22–24]. In Switzerland, a cross-sectional study revealed that in 2020, youths around 14 years old were 12 times more likely to spend over 4 h per day on electronic screens compared to their peers in 2012 [25]. This massive change in behavior led researchers to study the impact of screen use on adolescents’ health and document its deleterious effects on sleep [9]. Regarding physiological effects, between-subjects comparisons suggest that adolescents who use electronic screen devices more frequently during the day tend to experience worse sleep [10]. However, the same authors observed a weaker association in within-subject analyses, indicating that, at the individual level, using technology more on one day compared to another does not predict a later bedtime or shorter sleep duration [10]. To establish temporal order and potential causal relationships between screen time use and sleep outcomes at the intra-individual level, more robust experimental designs and advanced measurement techniques are required. A review by Hale et al. [26] found that, the majority of observational, but not experimental studies supported that screen time was negatively linked with sleep outcomes, such as shortened sleep duration and delayed bedtime. Yet, at the same time, we conducted an interventional study and demonstrated that stopping screen electronic devices after 9 p.m. for 2 weeks improved sleep duration and daytime alertness [27]. The negative impact of screen electronic devices on sleep can be explained by two different causes: (i) screen use is time-consuming and replaces sleeping time, and (ii) the strong emotional engagement (including social demands, stressful information and violent gaming, etc.) associated with the use of these screen electronic devices interferes with falling asleep quickly [26]. However, recent reviews and scientific consensus highlight a lack of evidence supporting a significant detrimental impact of blue light emitted by electronic screens on sleep. They also raise doubts about the arousing effects of electronic screen content. Instead, the observed effects appear to be more closely linked to sleep displacement and nighttime disruptions [28, 29].

The mere presence of screen electronic devices in adolescents’ bedrooms results in their heightened use before bedtime, that is then associated with later bedtimes and reduced sleep duration [30, 31]. This presence of devices does not help parents to monitor how their children use them [32]. The ability of the parents to structure their children’s behavior decreases as a function of the age in adolescence [33]. The few published studies on that topic reported that parental rules at home was associated with earlier schooldays bedtimes when controlling for age and gender [34], while conversely the absence of parental rules about the use of television, computer and video games was associated with later bedtimes [32]. Recent reviews have reaffirmed the positive impact of parental rules regarding the influence of electronic screen device use on sleep duration, quality, and bedtime habits [28, 35, 36]. However, these previous studies mainly focused on the presence or absence of parental rules regarding the use of screen electronic devices, irrespective of the type of rules implemented [37]. A gap in our understanding remains, as it is still unclear whether—and which—parental rules regarding screen use may positively influence adolescent sleep.

Based on the results from our initial intervention performed in 2015 [27], and from the studies reviewed above, we aimed at assessing the beneficial influence of parental control of screen use on sleep, as well as on academic outcomes [17]. Specifically, this study sought to more precisely identify whether certain parental rules regarding screen time could positively influence adolescents’ sleep duration and possibly school performance as well. To do so, we collected and analyzed data on the presence of specific parental rules.

## Materials and methods

### Population

We conducted the study in Geneva, Switzerland, on 356 healthy adolescents aged between 12 and 16 years old. We recruited adolescents via their schools and asked them to fill in several self-reported questionnaires in the classroom. We excluded participants who gave inconsistent and unreliable answers to the questionnaires such as a bedtime at 2 p.m. in the afternoon or being woken up by mosquitoes’ bites leading to a total sleep time of 2 h (*N* = 8). We also removed those with an age below 13 years old (*N* = 6) and those with an age above 15 years old (*N* = 13) to balance the number of responses across age groups. The analyses were thus performed on a final sample of 329 adolescents, aged between 13 and 15 (age, *M* = 14.2, *SD* = 0.74, 49% female participants).

We collected data on sleep and academic achievement, along with additional parental control variables (detailed below) for this sample.

### Sleep parameters

Sleep parameters including Sleep Onset Time (time at which participants usually fall asleep) and Waking-Up Time (time at which the participants usually wake up in the morning) were obtained via an online questionnaire. We computed the difference between Sleep Onset Time and Waking-Up Time to obtain the Total Sleep Time (TST). These questions were asked for both weekdays (“What time do you think you usually fall asleep on nights before school days?“) and weekends (“What time do you think you usually fall asleep on weekends, specifically Friday and Saturday nights?“), covering the entire school year, which lasted approximately 9 months. TST was then used as a dependent variable in our analyses. TST was separately estimated for pre-school nights during the week (Sunday to Thursday) and pre-weekend nights (Friday and Saturday).

### Parental rules

A whole section of the survey was dedicated to assessing the different types of parental rules about the use of screen electronic devices, all types included (phone, tablet, computer and TV), during weekdays’ evenings only. Adolescents were required to answer (yes/no) to six distinct questions. The initial one inquired about the presence or not of at least one rule set by their parents or caregiver, irrespective of the rule type (referred to as “Rule Present”) or none. Regardless of their response to this initial question, they were then given the chance to respond (yes/no) to five additional questions related to specific rules that they would “usually have to follow for screen electronic devices (phone, tablet, computer, TV) in the evenings before school”. Participants were classified as having “No rules at all” if they answered “No” to the initial general question and to all five specific rule items. However, if an adolescent answered “No” to the general question but “Yes” to at least one of the five specific rules, we still considered them to be subject to at least one parental rule. The first specific question was whether adolescents had to stop using screen electronic devices at a precise hour in the evening (e.g., stop at 9 p.m.) (“Fixed Hour”). The second was whether adolescents had a precise maximal time duration (e.g., 2 h) allowed on screen electronic devices (“Fixed Duration”). The third question concerned whether they were allowed to keep specifically their phone in the bedroom but on airplane mode (“Airplane Mode”). The fourth question was whether they had to leave specifically their phone outside of their bedroom during the night (“Out of Bedroom”). The fifth was whether they were forbidden from specifically using their phone in the evening (“No Phone”). To gain a qualitative perspective, we asked adolescents to rate the frequency of their last activity before falling asleep (e.g., reading a book, watching TV, etc.) on a scale ranging from “Never” to “Always.” The question was: “During the past two weeks, what was the last thing you did in bed just before falling asleep?”.

### Academic achievement

Academic achievement was assessed using the same questionnaire as the other variables. Adolescents were asked to report their overall school grade for the previous term. The exact question was “what was your overall average on your report card for the last term?”. In Switzerland, grades range from 0 (the lowest) to 6 (the highest).

### Procedure

Experimenters came to several high schools in the Geneva area and tested the adolescents in a computer classroom. There, each student was asked to fill in the online questionnaires, which started with demographic questions, then interrogations about sleep, mood, sleepiness, and parental rules. Prior to participation, adolescents’ informed consents were obtained from a parent and/or legal guardian. All data collected were kept anonymous using personal identification codes. This study was approved by local ethic committees (review board of the Geneva University Hospitals and Education Research Service, SRED) and was conducted in accordance with the Declaration of Helsinki.

### Statistical analyses

For all subsequent analyses, the level of significance was set to a *p*-value of < 0.05. Analyses were performed using RStudio (Version 1.4.1717). All variables were normally distributed. Paired t-tests and two-sample Welch’s t-tests were conducted as appropriate. Cohen’s d was calculated to report effect sizes for all t-tests.

We first examined the impact of parental rules on sleep by conducting two-sample t-tests on total sleep time (TST). We first tested the effect of the presence of any rule (“Rule Present”, i.e., at least one “Yes” answer to any of the questions about parental rules) on TST (in hours), with TST as the dependent variable and presence of any rule as dichotomous independent variable (no rule coded as 0; at least one rule coded as 1). We next performed separate analyses for each specific parental rule (Fixed Hour, Fixed Duration, Airplane Mode, Out of Bedroom, and No Phone), with TST as the dependent variable and each type of Parental Rule as dichotomous independent variables each coded as 0 (“No”) and 1 (“Yes”). We also performed a Multiple Linear Regression on TST including the five Parental Rules as predictors. Regarding the separate two-sample t-tests, we applied a Bonferroni correction as required.

Then, because previous reports suggested a relationship between sleep and academic achievement [38], we tested whether we could replicate such a correlation between sleep duration and academic achievement, but also investigate whether parental rules may be also involved.

## Results

### Links between parental rules and sleep duration

An examination of the demographic data related to the presence of multiple parental rules revealed that it is rare for adolescents in our sample to report being subject to more than one rule simultaneously. This trend is illustrated in the table below, which shows a decrease in the number of adolescents experiencing overlapping parental rules (see Table 1).

**Table 1:** Distribution of participants and TST means (in hours) by the number of reported parental rules

Number of parental rules simultaneously reported	TST mean (SD)	*N*
0	7.73 (1.22)	140
1	7.74 (1.18)	129
2	8.22 (0.99)	36
3	7.84 (1.49)	12
4	8.05 (0.12)	2
5	0	0

**Fig. 1:**
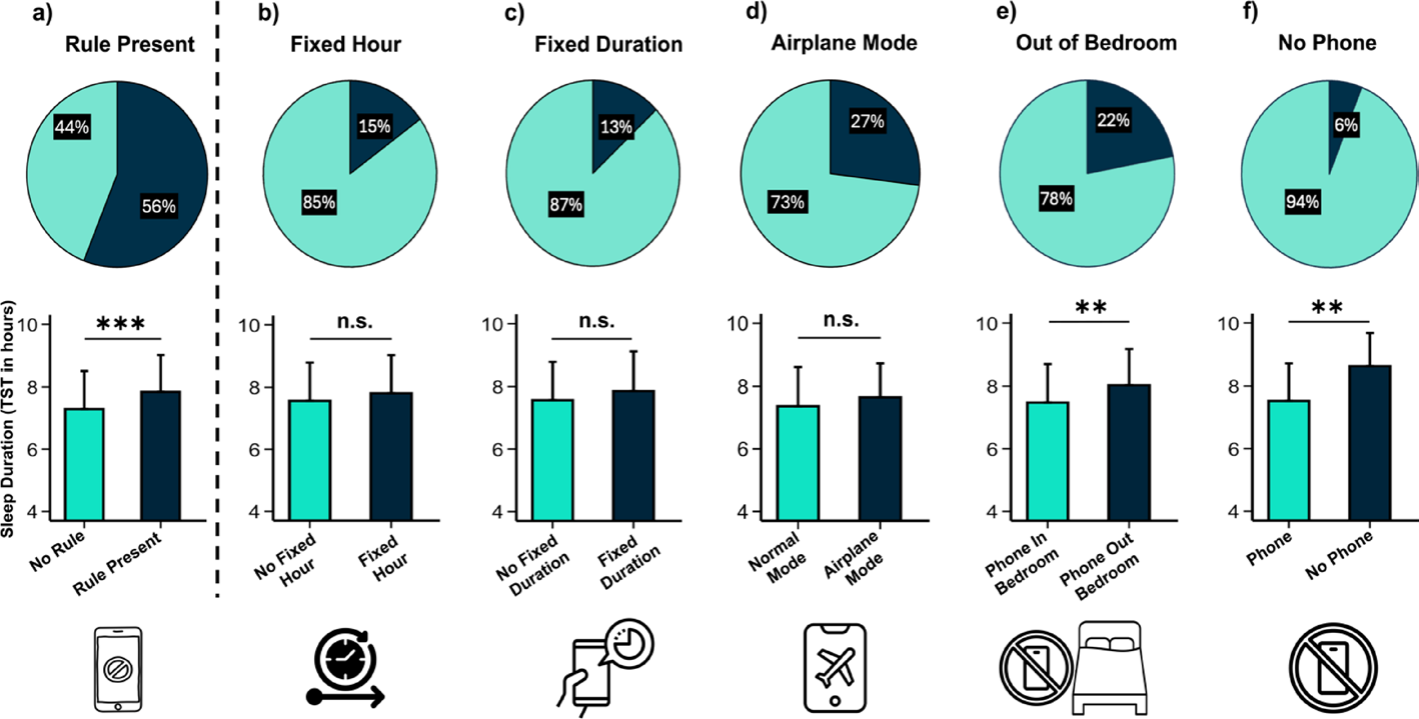
Distribution of the parental rules across the participants, and effect on sleep duration. On top: pie charts of the percentage of participants in each condition. On bottom: plots of the means of TST during the week nights as a function of the presence/absence of rules. With **a** Rule Present (*N* = 319), **b** Fixed Hour (*N* = 319), **c** Fixed Duration (*N* = 319), **d** Airplane Mode (*N* = 240), **e** Out of Bedroom (*N* = 319), **f** No Phone (*N* = 272). The different samples sizes are due to the exclusion of participants who generated incoherent answers and to missing data. Light Blue = rule not applied, Dark Blue = rule applied. Error bars = SD. Asterisks represent significance (*p*) of two-sample t-tests: ***< 0.001, **< 0.01 (Bonferroni correction for multiple testing).

Then, we statistically tested if the presence of parental rules about screen use in the evening affected sleep duration for the week nights, i.e., the nights most affected by sleep restriction in adolescents. A first two-sample t-test with TST during the week as the dependent variable and the presence of at least one parental rule (present vs. absent) as a categorical factor revealed a significant effect (t_(292.96)_ = −4.13, *p* <.001, d = − 0.47) (see Fig. 1a). Adolescents who had at least one parental rule reported, on average, sleeping 40 min more during the week, than those without any rules. To explore the role of specific parental rules, we then performed two-sample t-tests with TST as the dependent variable and each parental rule as categorical factors. Note that we looked at each parental rule regardless of the potential combination of several rules (e.g., having both a fixed hour and a fixed duration). To correct for multiple testing, we applied a Bonferroni correction. These analyses revealed that the following rules had no significant effect on TST: (i) Fixed Hour (stopping the use of electronic devices at a precise hour, for example stop at 9 p.m., t_(63.09)_ = 1.28, *p* = 1, d = 0.20) (see Fig. 1b), (ii) Fixed Duration (fixed maximal duration of time spent on screen electronic devices, for example 2 h, t_(317)_ = 1.39, *p* =.86, d = 0.24) (see Fig. 1c), and (iii) Airplane Mode (phone allowed in the bedroom on airplane mode, t_(127.83)_ = 1.73, *p* =.43, d = 0.24) (see Fig. 1 d). By contrast, TST significantly increased when the phone was not allowed in the bedroom (Out of Bedroom, t_(121.97)_ = −3.60, *p* =.002, d = − 0.47) (see Fig. 1e). On average, adolescents slept 33 min more when they were not allowed to keep their phones in their bedrooms. TST also increased during week nights when phone use was not allowed throughout the whole evening before going to bed (No Phone, t_(18.77)_ = 4.09, *p* =.003, d = 0.93) (see Fig. 1f), with a gain of 66 min on average. Note, however, that the presence of this rule was only reported by 17 adolescents out of the total 272 who answered this question, emphasizing the rare application of such a strict rule. Descriptive statistics on parental rules and the last activity performed before falling asleep are provided in the Supplementary Information (see Tables S1 and S2, Supplementary Information).

With the aim of characterizing the variance explained by each of the five parental rules on TST during the week, we computed a Multiple Linear Regression (see Table 2). Residuals were normally distributed, no multicollinearity effect was detected, and no outliers were identified by the Cook’s distance. The results indicated that when we considered the effect of all the rules together, only the exclusion of Out of Bedroom (see Fig. 1e) and No Phone during the evening (see Fig. 1f) significantly reduced the variance explained (see Table 2), thus confirming the significant influence of the two strictest rules only, as suggested by the two-sample t-tests described previously. Note that the tested model had a relatively small global effect size with an R^2^ around 9%.

**Table 2:** Multiple linear regression summary for TST during week including the five parental rules

	B	SE	β	SE	T value	*P* value
Fixed hour	0.23	0.20	0.07	0.06	1.20	0.23
Fixed duration	0.09	0.21	0.03	0.06	0.04	0.69
Airplane mode	0.28	0.18	0.10	0.06	1.60	0.11
Out of bedroom	0.55	0.18	0.19	0.06	3.11	**< 0.01** ^**a**^
No phone	0.88	0.30	0.18	0.06	2.93	**< 0.01** ^**a**^

^a^Bold *P* values represent statistically significant factors

### Links between parental rules, sleep duration and academic achievement

**Fig. 2:**
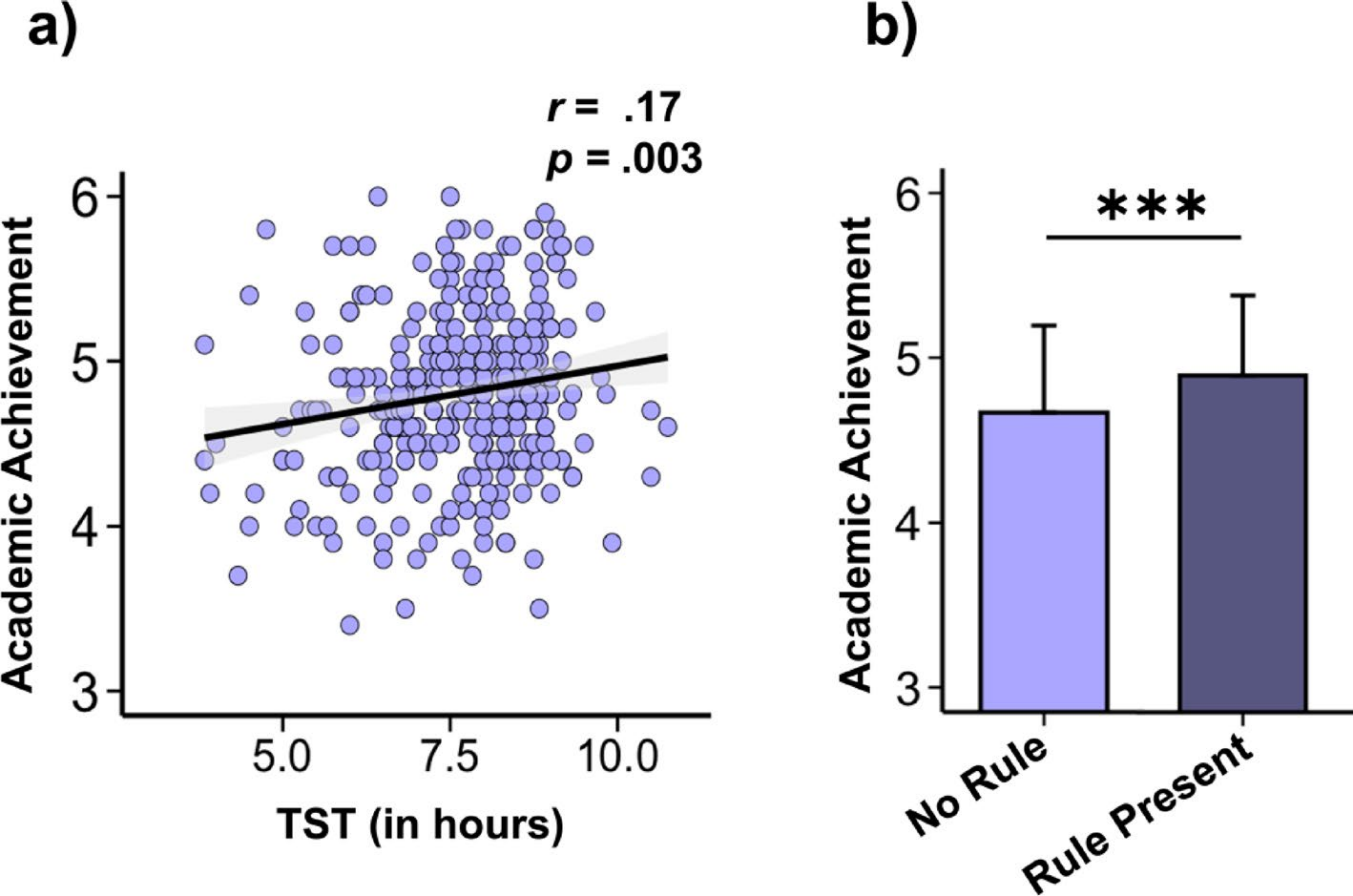
Associations between academic achievement, sleep duration, and the presence or absence of parental rules regarding the use of screen electronic devices. **a** Pearson correlation between TST (in hours) during the week and Academic Achievement (*N* = 308). **b** Bar plot of Academic Achievement depending on Rule Present (*N* = 319). Light Purple = rule not applied, Dark Purple = rule applied. Error bars = SD. Asterisks represent significance (*p*) of two-sample t-tests: ***< 0.001

Since TST was influenced by parental rules, we asked (i) whether TST during the week could also be linked with academic achievement and (ii) whether parental rules could have an effect on this variable. We also examined the differences in TST between weekdays and weekends and the association between this variability and academic achievement. To test these hypotheses, we firstly performed a Pearson correlation between sleep duration and Academic Achievement and obtained a positive link between them (*r* =.17, *p* =.03, CI _95%_ [0.06, 0.28]). Adolescents who indicated having longer TST during the week also reported higher Academic Achievement (see Fig. 2a). Then, we performed a two-sample t-test with academic achievement as the dependent variable and the presence of parental rules as the grouping variable, and we obtained a significant difference. Adolescents who reported having at least one rule showed better academic achievement compared to those without any rules (t_(284.26)_ = −3.91, *p* <.001, d = − 0.45) (see Fig. 2b). Finally, we conducted a paired t-test with TST as the dependent variable and weekday versus weekend as the grouping factor. The results revealed a significant difference, with adolescents sleeping on average one hour more on weekends compared to weekdays (t_(317)_ = −10.80, *p* <.001, d = − 0.61) (see Figure S1a, Supplementary Information). However, this variability between weekdays and weekends was not significantly associated with academic achievement (*r* =.002, *p* =.98, CI _95%_ [0.11, − 0.11]) (see Figure S1b, Supplementary Information).

## Discussion

This study clarifies the influence of parental rules concerning the use of electronic screen devices in the evening on sleep measures, as well as on academic outcomes in a sample of adolescents.

The main goal of the present study was to assess whether parental control over the use of electronic screen devices benefited sleep. We found that having at least one parental rule, regardless of the nature of the rule, was positively linked with longer sleep duration compared to having no rule whatsoever. Therefore, we confirmed our hypothesis regarding the beneficial role of parental control about electronic device use on sleep duration. When testing the impact of specific rules, our analyses revealed that not every parental rule on evening electronic screen device use (a critical time impacting sleep) exhibited a significant positive association with sleep duration. Specifically, only adolescents who were not allowed to keep their phone in their bedroom or to use their phone during the evening (the two strictest rules) reported a higher sleep duration as compared to those who were not imposed these restrictions. Other rules including stopping using screen electronic devices at a fixed hour (e.g., 9 p.m.) or after a fixed duration (e.g., after 2 h of use), as well as having the phone on airplane mode had no significant link with sleep duration. These findings suggest that implementing a rule requiring the physical removal of electronic screen devices from the bedroom is associated with longer sleep duration. In contrast, simply placing electronic screen devices on airplane mode or restricting their use to a certain duration or time while keeping it nearby does not appear to be sufficient to extend sleep duration. This may be explained by the fact that the mere proximity of the device, regardless of its operational mode, continues to serve as a source of attentional distraction and maintains accessibility to its various functions (e.g., social media, messaging). Having one’s electronic screen device nearby provides easy access, increasing the likelihood of using it and spending time on it. Consistent with previous research, the mere presence of a smartphone has been shown to impair cognitive performance and sleep, even when the device is not in active use ([35, 39, 40]). These results are also in line with those reported by Bower & Moyer [34] and with the review of Khor et al. [36] indicating a positive association between the presence of parental rules and both earlier bedtimes and longer sleep duration. Moreover, here we also found a positive impact on sleep duration of the presence of certain parental rules (the strictest ones) regarding phones specifically (unlike Pieters et al. [32]). Furthermore, the current study builds upon our previous experiment conducted by Perrault et al. [27], in which we observed that when adolescents were told to refrain from using screen electronic devices after 9 p.m., they obtained on average 17 min of additional sleep time. In this new survey, we found that adolescents whose caretakers applied stricter rules, such as removing the phone from the bedroom or preventing phone usage during the whole of the evening, reported sleeping an additional 33 and 66 min, respectively, compared to those without these parental regulations. These results are consistent with findings reported by Buxton et al. [41] regarding the relationship between having a phone present or absent in the bedroom and its impact on sleep duration. Our study however provides further insights into the specific parental rules associated with these behaviors. Please note that only a minority of families (22% and 6%, respectively) adhered to these most stringent rules. This highlights the importance of interpreting the small effect sizes with caution, as they may not be fully representative due to the limited number of adolescents contributing to the observed effects of these two rules. Moreover, longer sleep durations were associated with better academic outcomes; this was also the case when at least one parental rule was present. These results are in line with previous studies demonstrating that variability in sleep duration (i.e., represented by the person-standard deviation), measured by actigraphy, was associated with poorer academic outcomes in adolescents [42]. However, this effect needs to be confirmed in the future as a recent meta-analysis failed to find a significant association between sleep quantity and academic achievement [43]. Finally, we confirmed the presence of a sleep debt in adolescents, as they slept, on average, one hour more on weekends compared to weekdays [12]. However, the variability between weekdays and weekend was not associated with academic achievement. Future studies should therefore explore the relationship between this variability and more robust measures of daytime functioning. Overall, these findings support the significance of parental control over screen usage for subjectively reported higher sleep duration and school grades in adolescents. This study also highlights an urgent need to raise awareness and encourage families in their efforts to limit screen use in the evening. Caution is advised, as the sleep measures in our study were limited to sleep timing and did not assess other important aspects of sleep, such as quality or fragmentation, which parental rules might influence differently.

The first limitation of our study concerns the fact that the sleep parameters were derived from self-reported data, and not from objective measures such as actigraphy or electroencephalography (EEG). Yet, as reviewed by Ibáñez and colleagues [44], sleep diaries and sleep questionnaires show high reliability and sensitivity for assessing sleep quality and quantity, in addition to their low-cost and easy administration for at home data collection. Furthermore, as mentioned in the Methods section, we systematically excluded any participant with inconsistent data. A second limitation of this study is the absence of information about general parenting styles, family history of mental disorders, socio-economic status, stressful life events, and peer relations, thus precluding any investigation of these individual variables, which have been demonstrated to influence adolescents’ behavior, lifestyle, and mental health in prior research [45]. These variables could act as confounders, as we did not include covariates in our regression model. For instance, as adolescents grow older, the presence of parental rules tends to decline, along with overall parental control and authority [33]. Not including covariates represents a limitation to the validity of the current analyses. A third limitation concerns the lack of clarity surrounding the term “evening”. This term could have been better defined with a specific time frame (e.g., after 9 p.m.), particularly in relation to the strictest rule of not using phones at all. Similarly, future studies could benefit from evaluating the presence or absence of these parental rules on weekend nights to provide a more detailed analysis of adolescents’ sleep habits. A fourth limitation concerns the absence of subjective and/or objective measures of screen use. Specifically, we did not collect data on adolescents’ actual screen time, although we hypothesize that the beneficial impact of parental rules on sleep duration and academic achievement may be mediated by the regulation and reduction of screen use. Indeed, a recent meta-analysis has demonstrated a significant association between screen time and both sleep disturbances and lower academic achievement [46]. Future analyses should include this variable, either as a covariate or as a potential mediator. On the other hand, recent evidence also suggests a bidirectional relationship between the use of electronic screen devices and sleep. Indeed, sleep disruption may lead to increased use of electronic devices [47]. Additionally, it has been reported that adolescents use their screen electronic devices as a way to distract themselves from negative thoughts, potentially aiding sleep [48]. However, most recent experimental studies, meta-analyses and reviews indicate that the use of electronic screen devices tends to have a negative rather than a positive impact on sleep [31, 49, 50]. While our results revealed a strong association between parental control over screen use in the evening and sleep quantity, the absence of a rule regarding screen time may not necessarily imply that the adolescents are spending an excessive amount of time using their screen electronic devices and the other way around, the presence of a rule does not systematically ensure that teenagers are cautiously following them. Future experimental and longitudinal research is needed to identify the specific conditions under which using electronic screen devices could either benefit or harm sleep patterns. Similarly, future studies should account for the type of electronic devices targeted by the rules, the timing and proximity of rule enforcement to bedtime, and the degree to which these rules are consistently enforced at home. Finally, we acknowledge that parental rules about screen use in the evening may not be the sole factor influencing academic achievement. Indeed, families with the strictest evening rules are likely also strict about homework or school supervision, factors we did not measure here, and which are independent of the sleep schedule.

## Conclusion

Chronic sleep restriction in adolescents, is known to impede mental and academic status, and have major long-term consequences. Clarifying the relationship between sleep and the ubiquitous use of screen electronic devices is therefore critical for the development of strategies aiming at improving well-being in this young population. Parents seeking advice about sleep are most often left with little help. Here, we show that parental rules, in particular those imposing the device to be kept outside of the bedroom during the night, appear to be associated with better sleep quantity and academic outcomes, thus representing a potential effective tool for fighting against sleep curtailment, and its affective and cognitive adverse consequences. We therefore suggest that such accessible, science-informed recommendations for the adolescents and their parents, as well as interventions in the classroom, including workshops, may prevent or limit a major and increasing public health issue by enhancing sleep quality and quantity in adolescents.

## Supplementary Information

Supplementary Material 1

## Data Availability

The data that support the findings of this study are not openly available due to reasons of sensitivity and are available from the corresponding author upon reasonable request. Contact: Kevin.Mammeri@unige.ch.

## References

[1] Born J, Rasch B, Gais S. Sleep to remember. Neuroscientist. 2006;12(5):410–24. 16957003 10.1177/1073858406292647

[2] Sterpenich V, Ceravolo L, Schwartz S. Sleep deprivation disrupts the contribution of the hippocampus to the formation of novel lexical associations. Brain and Language. 2017;167:61–71. 28173964 10.1016/j.bandl.2016.12.007

[3] Hudson AN, Van Dongen HPA, Honn KA. Sleep deprivation, vigilant attention, and brain function: a review. Neuropsychopharmacology. 2020;45(1):21–30. 10.1038/s41386-019-0432-6. 31176308 10.1038/s41386-019-0432-6 PMC6879580

[4] Palmer CA, Oosterhoff B, Bower JL, Kaplow JB, Alfano etCA. Associations among adolescent sleep problems, emotion regulation, and affective disorders: Findings from a nationally representative sample. J Psychiatr Res. 2018;96:1–8. 28941378 10.1016/j.jpsychires.2017.09.015

[5] Scott J, Kallestad H, Vedaa O, Sivertsen B, Etain B. Sleep disturbances and first onset of major mental disorders in adolescence and early adulthood: A systematic review and meta-analysis. Sleep Med Rev. 2021;57:101429. 10.1016/j.smrv.2021.101429. 33549912 10.1016/j.smrv.2021.101429

[6] Hirshkowitz M, et al. National sleep foundation’s sleep time duration recommendations: methodology and results summary. Sleep Health. 2015;1(1):40–3. 10.1016/j.sleh.2014.12.010. 29073412 10.1016/j.sleh.2014.12.010

[7] Paruthi S, et al. Consensus statement of the American academy of sleep medicine on the recommended amount of sleep for healthy children: methodology and discussion. J Clin Sleep Med. 2016;12(11):1549–61. 10.5664/jcsm.6288. 27707447 10.5664/jcsm.6288 PMC5078711

[8] Seton C, Fitzgerald DA. Chronic sleep deprivation in teenagers: practical ways to help. Paediatr Respir Rev. 2021;40:73–9. 10.1016/j.prrv.2021.05.001. 34144910 10.1016/j.prrv.2021.05.001

[9] Kiss O et al. Effects of the COVID-19 pandemic on screen time and sleep in early adolescents. Health Psychol*. *2023; 42(12): 894–903. 10.1037/hea0001251 10.1037/hea0001251 PMC10522787 36972087

[10] Burnell K, George MJ, Jensen M, Hoyle RH, Odgers CL. Associations between adolescents’ daily digital technology use and sleep. J Adolesc Health. 2022;70(no 3):450–6. 10.1016/j.jadohealth.2021.09.033. 34756778 10.1016/j.jadohealth.2021.09.033 PMC8860860

[11] Albrecht JN, Werner H, Rieger N, Jenni OG, et, Huber R. Sleep and health-related characteristics among adolescents during covid-19 an update. IJERPH (2022); 19(9): 5078. 10.3390/ijerph19095078 10.3390/ijerph19095078 PMC9105238 35564473

[12] Galland BC et al. Establishing normal values for pediatric nighttime sleep measured by actigraphy: a systematic review and meta-analysis. Sleep 41(4). 10.1093/sleep/zsy017 10.1093/sleep/zsy017 29590464

[13] Minges KE et, Redeker NS. Delayed school start times and adolescent sleep: a systematic review of the experimental evidence. Sleep Med Rev. 2016; 28: 2886–95. 10.1016/j.smrv.2015.06.002 10.1016/j.smrv.2015.06.002 PMC4844764 26545246

[14] Gariepy G, et al. How are adolescents sleeping? Adolescent sleep patterns and sociodemographic differences in 24 European and North American countries. J Adolesc Health. 2020;66(6):S81–8. 10.1016/j.jadohealth.2020.03.013. 32446613 10.1016/j.jadohealth.2020.03.013

[15] Jahrami H, et al. Sleep quality worsens while perceived stress improves in healthcare workers over two years during the COVID-19 pandemic: results of a longitudinal study. Healthcare. 2022;10(8):1588. 10.3390/healthcare10081588. 36011245 10.3390/healthcare10081588 PMC9408655

[16] Matricciani LA, Olds TS, Blunden S, Rigney G, Williams MT. Never enough sleep: a brief history of sleep recommendations for children. Pediatrics. 2012;129(no 3):548–56. 10.1542/peds.2011-2039. 22331340 10.1542/peds.2011-2039

[17] Sivertsen B, Glozier N, Harvey AG, et, Hysing M. Academic performance in adolescents with delayed sleep phase. Sleep Med.* 2015: *16(9): 1084–1090. 10.1016/j.sleep.2015.04.011 10.1016/j.sleep.2015.04.011 26298783

[18] Affuso G, et al. The effects of teacher support, parental monitoring, motivation and self-efficacy on academic performance over time. Eur J Psychol Educ. 2023;38(1):1–23. 10.1007/s10212-021-00594-6.

[19] Dimitriou D, Le Cornu Knight F, et, Milton P. The Role of Environmental Factors on Sleep Patterns and School Performance in Adolescents. *Front. Psychol.*2015; 6. 10.3389/fpsyg.2015.01717 10.3389/fpsyg.2015.01717 PMC4664801 26648878

[20] Dewald JF, Meijer AM, Oort FJ, Kerkhof GA, Bögels SM. The influence of sleep quality, sleep duration and sleepiness on school performance in children and adolescents: a meta-analytic review. Sleep Med Rev. 2010;14(3):179–89. 10.1016/j.smrv.2009.10.004. 20093054 10.1016/j.smrv.2009.10.004

[21] Crede J, Wirthwein L, McElvany N, et, Steinmayr R. Adolescentsâ€™ academic achievement and life satisfaction: the role of parentsâ€™ education. Front Psychol. 2015. 6. 10.3389/fpsyg.2015.00052 10.3389/fpsyg.2015.00052 PMC4315030 25691877

[22] Rideout V et, Robb MB. The common sense census: media use by tweens and teens. 2019. [En ligne]. Disponible sur: https://www.commonsensemedia.org/research/the-common-sense-census-media-use-by-tweens-and-teens-2019

[23] Rideout V et, Robb MB. The common sense census: media use by tweens and teens. 2021. [En ligne]. Disponible sur: https://www.commonsensemedia.org/research/the-common-sense-census-media-use-by-tweens-and-teens-2019

[24] Nagata JM, et al. Screen time use among US adolescents during the COVID-19 pandemic: findings from the adolescent brain cognitive development (ABCD) study. JAMA Pediatr. 2022;176(1):94–6. 10.1001/jamapediatrics.2021.4334. 34724543 10.1001/jamapediatrics.2021.4334 PMC8561427

[25] Mora-Monteros M, Suris J-C, Chok L, Siwiak A, Stadelmann S, Barrense-Dias Y. Evolution of screen use among youth between 2012 and 2020 in Switzerland. Arch Pediatr. 2023;30(8):563–6. 10.1016/j.arcped.2023.09.001. 37798215 10.1016/j.arcped.2023.09.001

[26] Hale L, Li X, Hartstein LE, LeBourgeois MK. Media use and sleep in teenagers: what do we know? Curr Sleep Medicine Rep. 2019;5(3):128–34. 10.1007/s40675-019-00146-x.

[27] Perrault AA et al. Reducing the use of screen electronic devices in the evening is associated with improved sleep and daytime vigilance in adolescents. Sleep*. *2019*: *42(9);1-10. 10.1093/sleep/zsz125 10.1093/sleep/zsz125 31260534

[28] Bauducco S, Pillion M, Bartel K, Reynolds C, Kahn M, et, Gradisar M. A bidirectional model of sleep and technology use: a theoretical review of how much, for whom, and which mechanisms. Sleep Med Rev*. *2024: 76; 101933. 10.1016/j.smrv.2024.101933 10.1016/j.smrv.2024.101933 38657359

[29] Hartstein LE, et al. The impact of screen use on sleep health across the lifespan: a National Sleep Foundation consensus statement. Sleep Health. 2024;10(4):373–84. 10.1016/j.sleh.2024.05.001. 38806392 10.1016/j.sleh.2024.05.001 PMC13181348

[30] Hale L et al. Youth Screen Media Habits and Sleep. Child and Adolescent Psychiatric Clinics of North America*. *2018: 27(2); 229–45. 10.1016/j.chc.2017.11.014 10.1016/j.chc.2017.11.014 PMC5839336 29502749

[31] Yu DJ, Wing YK, Li TMH, Chan NY. The impact of social media use on sleep and mental health in youth: a scoping review. Curr Psychiatry Rep. 2024. 10.1007/s11920-024-01481-9. 38329569 10.1007/s11920-024-01481-9 PMC10948475

[32] Pieters D, et al. Effects of pre-sleep media use on sleep/wake patterns and daytime functioning among adolescents: the moderating role of parental control. Behav Sleep Med. 2014;12(6):427–43. 10.1080/15402002.2012.694381. 24617896 10.1080/15402002.2012.694381

[33] Spitz A, Winkler Metzke C, Steinhausen H-C. Growth trajectories of perceived parental behavior during adolescence. Child Psychiatry Hum Dev. 2021;52(6):1154–63. 10.1007/s10578-020-01095-1. 33170413 10.1007/s10578-020-01095-1

[34] Bowers JM et, Moyer A. Adolescent sleep and technology use rules results from the California health interview survey. Sleep Health. 2020; 6(1): 19–22. https://doi.org/10.1016/j.sleh.2019.08.011. 10.1016/j.sleh.2019.08.011 10.1016/j.sleh.2019.08.011 PMC7346706 31732441

[35] Carter B, Rees P, Hale L, Bhattacharjee D. et M. S. Paradkar. Association between portable screen-based media device access or use and sleep outcomes: a systematic review and meta-analysis. JAMA Pediatr. 2016; 6 (1): 19–22. 10.1001/jamapediatrics.2016.2341 10.1001/jamapediatrics.2016.2341 PMC5380441 27802500

[36] Khor SPH, McClure A, Aldridge G, Bei B, Yap etMBH. Modifiable parental factors in adolescent sleep: a systematic review and meta-analysis. Sleep Med Rev*. *2021; 56: 101408. 10.1016/j.smrv.2020.101408 10.1016/j.smrv.2020.101408 33326915

[37] Giovanelli A, Ozer EJ, Adams SH, Park MJ. et E. M. Ozer,. Adolescent Technology-use Rules and Sleep in a Large Representative Sample. J Adolesc Health. 2022; 70 (4):682–5. 10.1016/j.jadohealth.2021.10.025 10.1016/j.jadohealth.2021.10.025 PMC11925058 34991931

[38] Hysing M, Harvey AG, Linton SJ, Askeland KG, Sivertsen B. Sleep and academic performance in later adolescence: results from a large population-based study. J Sleep Res. 2016;25(3):318–24. 10.1111/jsr.12373. 26825591 10.1111/jsr.12373

[39] Niu G, Shi X, Zhang Z, Yang W, Jin S, Sun X. Can smartphone presence affect cognitive function? The moderating role of fear of missing out. Comput Hum Behav. 2022;136:107399. 10.1016/j.chb.2022.107399.

[40] Skowronek J, Seifert A, et, Lindberg S. The mere presence of a smartphone reduces basal attentional performance. Sci Rep. 2023; 13(1): 9363. 10.1038/s41598-023-36256-4 10.1038/s41598-023-36256-4 PMC10249922 37291199

[41] Buxton OM, Chang A-M, Spilsbury JC, Bos T, Emsellem H, Knutson KL. Sleep in the modern family: protective family routines for child and adolescent sleep. Sleep Health. 2015;1(1):15–27. 10.1016/j.sleh.2014.12.002. 26779564 10.1016/j.sleh.2014.12.002 PMC4712736

[42] Mathew GM, Reichenberger DA, Master L, Buxton OM, Chang A-M, Hale etL. Actigraphic sleep dimensions and associations with academic functioning among adolescents. Sleep 2024; 47 (7): 62. 10.1093/sleep/zsae062 10.1093/sleep/zsae062 PMC11236952 38442263

[43] Musshafen LA, et al. Associations between sleep and academic performance in US adolescents: a systematic review and meta-analysis. Sleep Med. 2021;83:71–82. 10.1016/j.sleep.2021.04.015. 33991893 10.1016/j.sleep.2021.04.015

[44] Ibáñez V, Silva J, Cauli O. A survey on sleep questionnaires and diaries. Sleep Med. 2018;42:90–6. 10.1016/j.sleep.2017.08.026. 29458752 10.1016/j.sleep.2017.08.026

[45] Basu S et, Banerjee B. Impact of environmental factors on mental health of children and adolescents: A systematic review. Children and Youth Services Rev. 2020; 119: 105515. 10.1016/j.childyouth.2020.105515

[46] Gull M. Ruth sravani. Do screen time and social media use affect sleep patterns, psychological health, and academic performance among adolescents Evidence from bibliometric analysis. Child Youth Serv Rev. 2024;164:107886. 10.1016/j.childyouth.2024.107886.

[47] Mazzer K, Bauducco S, Linton SJ, et, Boersma K. Longitudinal associations between time spent using technology and sleep duration among adolescents. *J Adolesc. *2018; 66(1): 112-9. 10.1016/j.adolescence.2018.05.004 10.1016/j.adolescence.2018.05.004 29842997

[48] Daniels A et al. Technology use as a sleep-onset aid: are adolescents using apps to distract themselves from negative thoughts. Sleep Adv*.* 2023; 4(1): 47. 10.1093/sleepadvances/zpac047 10.1093/sleepadvances/zpac047 PMC10108637 37193290

[49] Pillion M, Gradisar M, Bartel K, Whittall H, et, Kahn M. What’s app-ning to adolescent sleep? links between device, app use, and sleep outcomes. Sleep Medicine. 2022; 100: 174-82. 10.1016/j.sleep.2022.08.004 10.1016/j.sleep.2022.08.004 36084495

[50] Richter SA, Ferraz-Rodrigues C, Schilling LB, Camargo NF, et, Nunes ML. Effects of the COVID ‐19 pandemic on sleep quality in children and adolescents: a systematic review and meta‐analysis. J Sleep Res. 2023; 32(1). 10.1111/jsr.13720 10.1111/jsr.13720 PMC9539085 36000251

