# Supplementary material for: Influence of parental rules about screen electronic device use in the evening on sleep in adolescents
Source: Discov Public Health. 2025 Sep 6;22(1):517. doi: 10.1186/s12982-025-00923-w (PMC12414006; doi:10.1186/s12982-025-00923-w)
Supplement: Supplementary file 1 — Supplementary Material 1 [file 12982_2025_923_MOESM1_ESM.docx]

**Supplementary Information**

Influence of parental rules about screen electronic device use in the evening on sleep in adolescents

Kevin Mammeri^1,2,*^, Laura Riontino^1,2^, Sophie Schwartz^1,2^ & Virginie Sterpenich^1,2,*^

^1^Department of Basic Neurosciences, University of Geneva, Switzerland

^2^Swiss Center for Affective Science, Geneva, Switzerland,

*Corresponding authors. Kevin Mammeri, Campus Biotech, Chemin des Mines, 9, 1202 Geneva, Switzerland. Email: Kevin.Mammeri@unige.ch; Virginie Sterpenich. Email: Virginie.Sterpenich@unige.ch.

**Complete list of authors**:

Mammeri, Kevin, University of Geneva, Department of Basic Neurosciences, E-mail address: Kevin.Mammeri@unige.ch

Riontino, Laura, University of Geneva, Department of Basic Neurosciences, E-mail address: Laura.Riontino@unige.ch

Schwartz, Sophie, University of Geneva, Department of Basic Neurosciences, E-mail address: Sophie.Schwartz@unige.ch

Sterpenich, Virginie, University of Geneva, Department of Basic Neurosciences, E-mail address: Virginie.Sterpenich@unige.ch


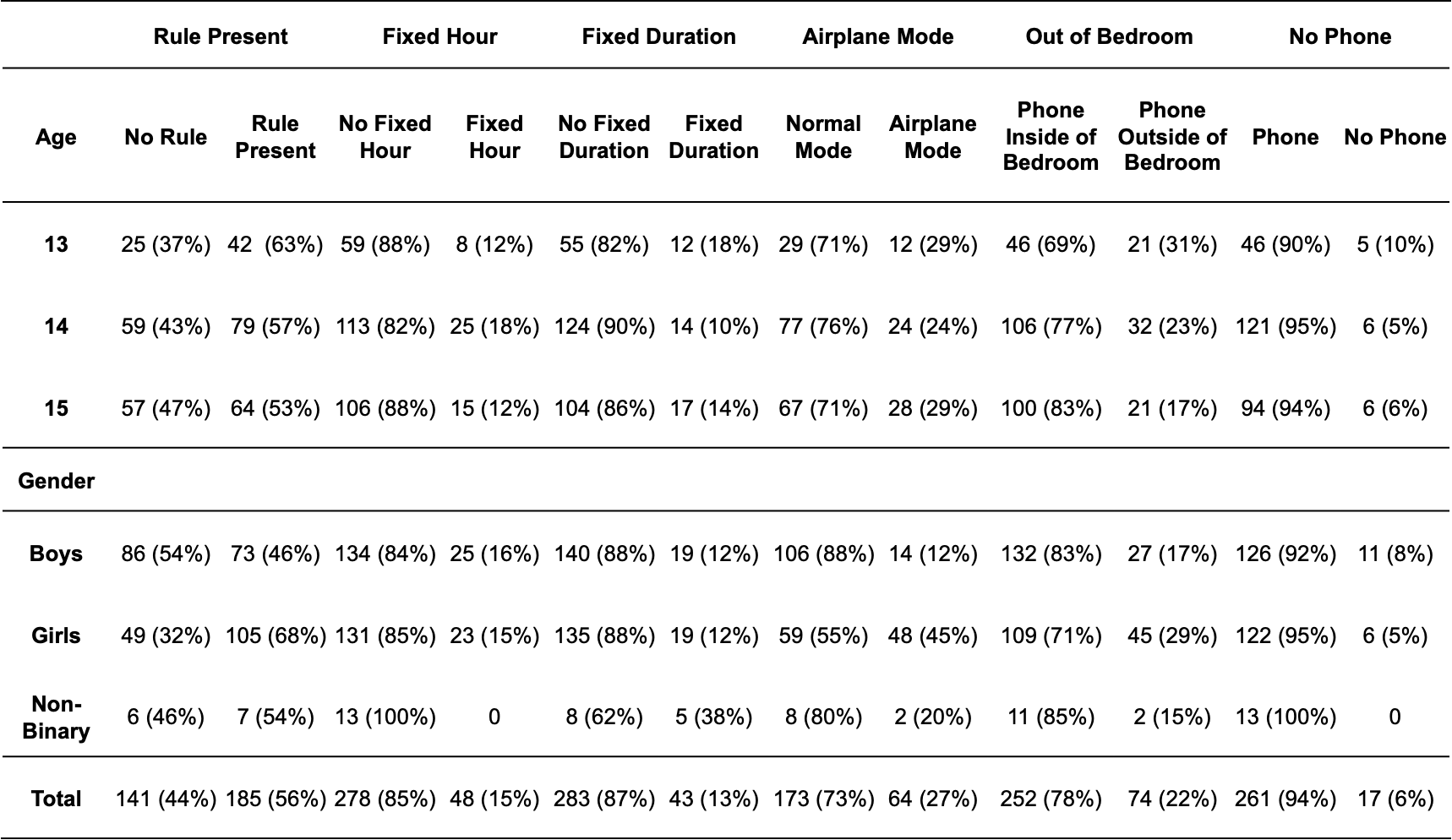
**Table S1**. Descriptive statistics of Parental Rules depending on Age and Gender.


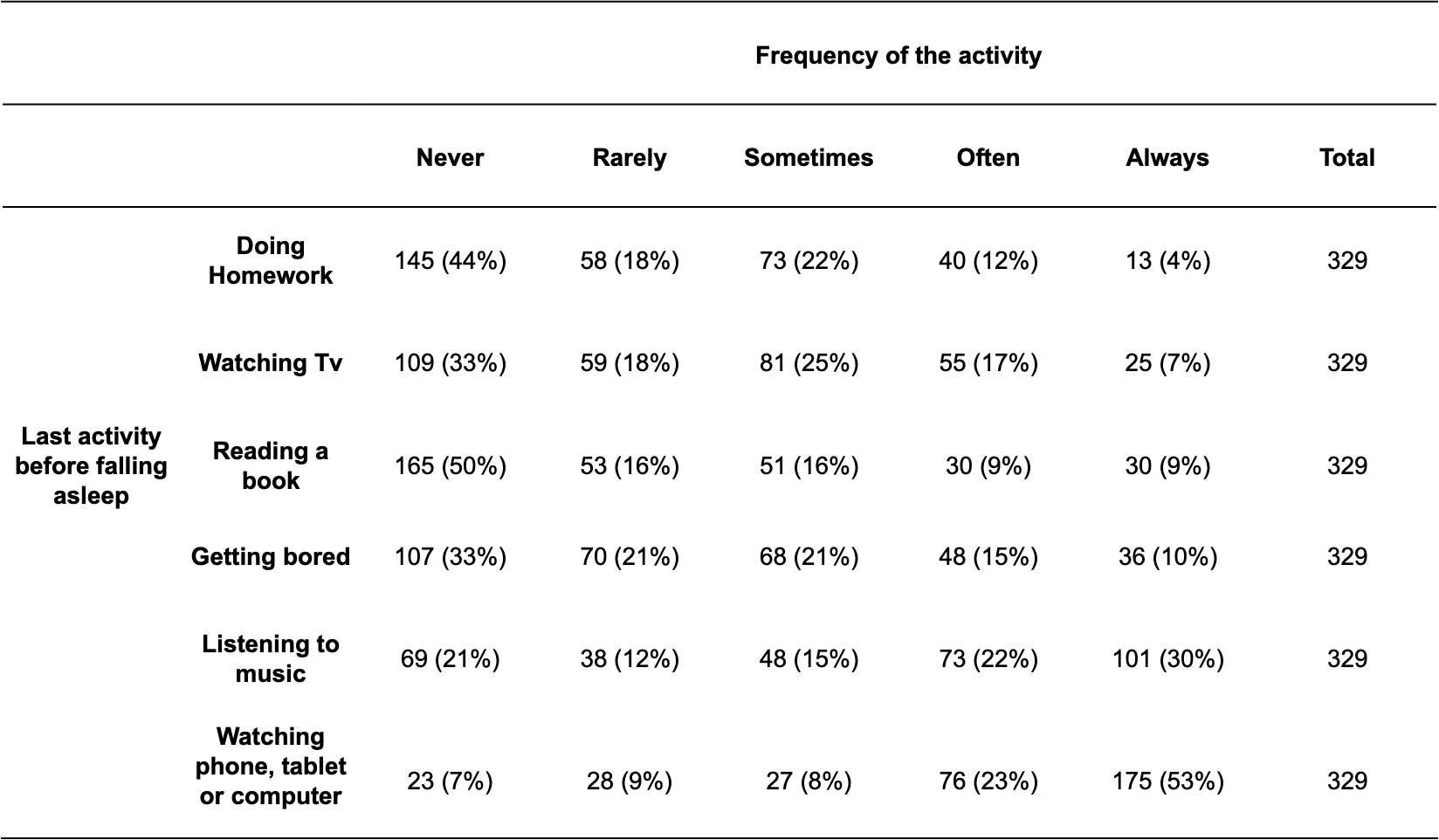
**Table S2**. Descriptive statistics of answers to the question “During the past two weeks, what was the last thing you did in bed just before falling asleep”.

**
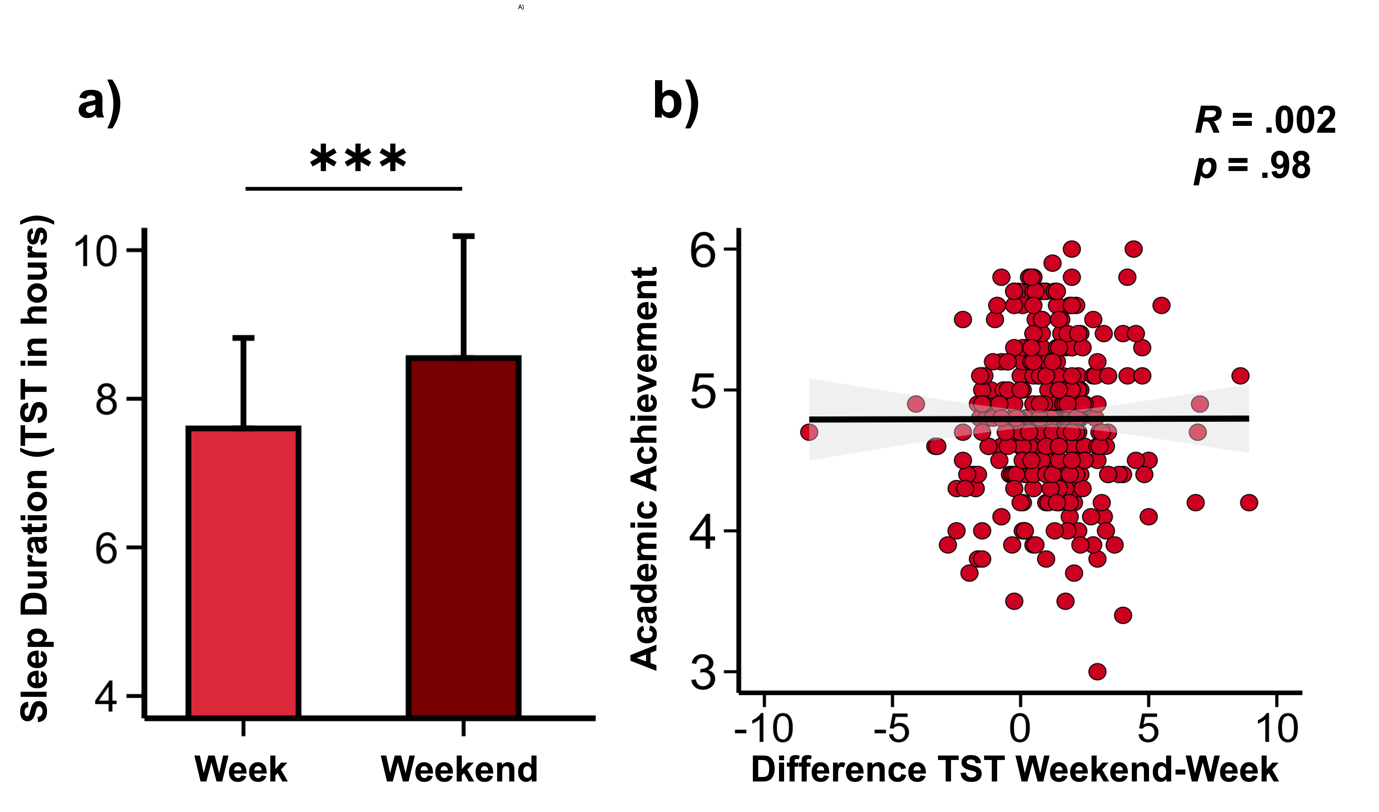
**

**Figure S1**. Investigation of sleep duration (TST) and Academic Achievement depending on the weekdays versus weekends. between TST Weekend and TST Week. (a) Paired t-test between TST reported during weekdays (Week) and weekends (Weekend). (b) Pearson correlation between Academic Achievement and the difference between TST Weekend and TST Week as a measure of the variability. Light Red = Week, Dark Red = Weekends. Error bars = SD. Asterisks represent significance (*p*) of dependent t-test: ***< .001
